# Low abundance of phytophagous nematodes under invasive exotic *Pinus elliottii* – enemy release and plant–soil feedbacks

**DOI:** 10.1111/nph.70852

**Published:** 2025-12-28

**Authors:** Lynda S. C. Guerrero, Erika Buscardo, Mario M. Inomoto, Laszlo Nagy

**Affiliations:** ^1^ Department of Animal Biology University of Campinas Campinas SP 13083‐862 Brazil; ^2^ Ecology Post‐graduate Programme, Institute of Biology University of Campinas Campinas SP 13083‐862 Brazil; ^3^ Ecology Post‐Graduate Programme, Department of Ecology Federal University of Rio Grande do Norte Natal RN 59078‐970 Brazil; ^4^ Department of Plant Pathology and Nematology, Luiz de Queiroz College of Agriculture University of São Paulo Piracicaba SP 13418‐900 Brazil

**Keywords:** Atlantic Forest biogeographic domain, enemy release hypothesis, montane ecosystems, *Pinus elliottii*, plant–soil feedbacks, soil nematode communities

## Abstract

- According to the enemy release hypothesis (ERH), the fitness of exotic plants and their capacity to become invasive in their area of introduction may partly be attributable to the loss of their natural enemies. Invasive species may also benefit from modifying soil attributes and thereby creating a positive soil–plant feedback.
- We assessed the relationship between time since the establishment of the invasive *Pinus elliottii* and enemy release in a montane pine–nematode‐specific context within the Atlantic Forest domain, by comparing soil nematode communities/functional diversity along a virtual chronosequence of invasion.
- Our findings confirmed the premises of the ERH and suggest that invasion may be facilitated by a lesser nematode load on pine compared to that on native species. The impact of *P. elliottii* on nematode communities over time was mainly driven by changes in the trophic structure with a major depletion of phytophagous species and overall nematode richness.
- The findings suggest that *P. elliottii* after experiencing an initial reduction in natural enemy pressure in its exotic range, further changes the composition of soil organisms in its rhizosphere. This has implications for plant–soil feedbacks which, in turn, affect the dynamics of pine invasion in neotropical montane ecosystems.

## Resumen

- De acuerdo con la hipótesis de liberación de enemigos naturales (ERH, por sus siglas en inglés), la retroalimentación de las plantas exóticas y su capacidad de volverse invasoras en su área de introducción puede atribuirse parcialmente a la pérdida de sus enemigos naturales. Las especies invasoras también pueden beneficiarse de la modificación de los atributos del suelo y así crear una retroalimentación positiva suelo‐planta.
- Comparamos la relación entre el tiempo desde el establecimiento de la invasora *Pinus elliottii* y el escape de enemigos naturales en un contexto específico de pino–nemátodo en un ambiente montano dentro del dominio del Bosque Atlántico, mediante la comparación de las comunidades de nemátodos del suelo/diversidad funcional a partir de una cronosecuencia virtual de invasión.
- Nuestros resultados confirman los postulados de la ERH y sugieren que la invasión puede ser facilitada por una menor carga de nemátodos en pinos en comparación con las especies nativas. El impacto de *P. elliottii* en las comunidades de nemátodos con el tiempo fue principalmente determinado por cambios en la estructura trófica con una marcada reducción de especies de fitófagos y riqueza total de nemátodos.
- Los resultados sugieren que *P. elliottii* tras experimentar una reducción inicial en la presión de enemigos naturales en su área de distribución exótica, también modifica la composición de los organismos del suelo en su rizosfera. Esto tiene implicaciones para la retroalimentación planta‐suelo, lo que a su vez afecta las dinámicas de la invasión del pino en los ecosistemas neotropicales de montaña.

## Introduction

The success of plant introductions has partly been explained by the presumed escape of introduced plants in their new environment from their natural enemies (e.g. herbivores, pathogens) that occur in their natural range (viz. enemy release hypothesis – ERH; Keane & Crawley, 2002) and which results in a reduced enemy load. The likelihood and strength of enemy release, depends on specific contexts, including phylogenetic relatedness of exotic and native species, time since introduction and resource levels (Keane & Crawley, 2002; Blumenthal, 2006; Mitchell *et al*., 2006; Hawkes, 2007; Brian & Catford, 2023).

In addition to the reduction in natural enemy pressure, the exotic species may further modify soil attributes/organisms in their rhizospheres (e.g. the composition and structure of soil communities via new biochemistry). According to the ‘novel weapon’ hypothesis (Callaway & Ridenour, 2004), exotic plant species may also produce defense compounds that are not produced by species of the invaded community that could further reduce the abundance and/or richness of potential enemies, including plant‐feeding nematodes (Callaway *et al*., 2008; Schaffner *et al*., 2011).

This affects, in turn, the plants via positive soil biota–plant feedback (Klironomos, 2002; Reinhart *et al*., 2003; van der Putten *et al*., 2005; Suding *et al*., 2013; Dickie *et al*., 2017a; Wilschut *et al*., 2019). Whether feedbacks are positive or negative depends on the balance between relative positive effects of beneficial soil organisms (e.g. mycorrhizas, nitrogen‐fixing bacteria) and the relative negative effects of plant enemies (e.g. soil‐borne pathogens, herbivores) as well as the indirect effect that plant–soil biota interactions have on plant–plant interactions (Reinhart & Callaway, 2006).

Introduced species may therefore improve their fitness (population growth/reproductive potential) via a reduction in fitness limitation that their natural enemies cause in their home range and via the benefit that they potentially derive from altering soil chemistry and nutrient cycling and modifying plant–soil feedbacks in their exotic range (Keane & Crawley, 2002; Callaway *et al*., 2004; Wubs *et al*., 2019; Aldorfová *et al*., 2020). This, in the absence of dispersal limitation, could increase the spread from the site of introduction to neighbouring habitats, such as has been the case of several species of the genus *Pinus* introduced in the Southern Hemisphere (Simberloff *et al*., 2010), where, except for *P. merkusii* Jungh. & Vriese in south‐east Asia, no native species belonging to Pinaceae exist.

A recent census has shown that plantations of *Pinus* spp. in Brazil cover *c*. 1.6 M ha, most of it within the Atlantic Forest biogeographic domain (Industria Brasileira de Árvores, 2020). The plantations have replaced native vegetation, dominated by arbuscular mycorrhizal (AM) symbionts, with monospecific exotic pine species that form ectomycorrhizal (ECM) associations (Wilson & Mayle, 2024). Additionally, plantations have served as source populations of invasion (e.g. *P. elliottii* Engelm., *P. patula* Schtdl. & Cham., and *P. taeda* L.) of montane grasslands (Milani *et al*., 2020), rich in endemic species, including protected areas in the Mantiqueira mountain range, south‐eastern Brazil. The establishment of pine species has been changing the structure and ecology of these native ecosystems (Taylor *et al*., 2016; Moyano *et al*., 2023).

Pine–fungal symbiont co‐invasion can have major effects on ecosystem properties such as soil nutrient cycling (e.g. recalcitrant litter and root exudates; Chapela *et al*., 2001; Reich *et al*., 2005), community composition of soil biota and on soil functions, either through modified resource inputs and/or through changes in biotic interactions (Dickie *et al*., 2011, 2014, 2017b; Peralta *et al*., 2019; Sapsford *et al*., 2022). Soil communities mediate plant–soil feedbacks between non‐native and native species, also described for processes during pine invasion (Nuske *et al*., 2021; Green *et al*., 2025; Dudenhöffer & Hulme, 2025a,b). Pines depend on ECM fungi for successful establishment and spread and the role of fungal symbionts in pine invasion has received significant attention (Nuñez *et al*., 2009; Dickie *et al*., 2010; Policelli *et al*., 2019, 2023). In contrast to the positive effects of beneficial ECM fungi, the potential role played by soil‐borne enemies on pine invasion and the feedback of enemy release on plant–soil interaction has been less investigated.

Nematodes are important soil‐borne enemies that could directly (e.g. damage to plant tissues) and indirectly (e.g. changes in trophic interactions) affect the invasiveness of exotic pine species in the Southern Hemisphere (Thakur & Geisen, 2019). Nematodes are the most abundant and functionally diverse soil animals (van den Hoogen *et al*., 2019). They are involved in soil processes including decomposition and nutrient cycling, and affect plant growth (Ingham *et al*., 1985; Osler & Sommerkorn, 2007; Neher *et al*., 2012). Based on their feeding habits they have been classified into phytophagous species (henceforth also referred to as plant‐ or root‐feeding nematodes), bacterivores, omnivores, fungivores, algivores and predators (Yeates *et al*., 1993).

Root‐feeding nematodes cause tissue necrosis, gall formation and inhibition of root growth (Ruehle, 1973) and their presence and/or activity predispose plants to be attacked by opportunistic pathogens such as fungi (Castillo *et al*., 1998; Back *et al*., 2002; Jones *et al*., 2013; Wheeler *et al*., 2019). The ecological interactions of nematodes with their hosts and with other consumers of the soil food web ultimately affect plant performance and distribution (Neher, 2010; Topalović & Geisen, 2023). Damage caused by phytophagous nematodes to forest trees generally results in nonspecific aboveground symptoms to the plant, making it difficult to quantify nematode effects in the field (Singh *et al*., 2015; Khan *et al*., 2020). Phytophagous nematodes reduce seedling growth (e.g. *Rotylenchus* spp.; Magnusson, 1983) and cause chlorosis and death of pine seedlings in nurseries (e.g. species of *Belonolaimus*, *Hoplolaimus*, *Pratylenchus*, *Tylenchorhynchus* and *Trichodorus*; Ruehle, 1966, 1968). Species of some genera, including *Criconemoides*, *Hemicycliophora*, and *Helicotylenchus* parasitise and reproduce on *P. elliottii* (Khan, 2012).

The diversity and abundance of nematode communities have been shown to be negatively impacted by exotic pine species (Yeates & Saggar, 1998; Scholes & Nowicki, 2000; Dehlin *et al*., 2008). It remains poorly known, however, if invasive pine species experience enemy release from soil phytophagous nematodes and if soil enemy abundance/diversity changes at different stages of the invasion pathway. Quantifying the relationship between time since invasive pine establishment and the abundance and diversity of phytophagous nematodes in montane ecosystems within the Atlantic Forest domain would contribute to a better understanding of invasion dynamics.

Using as a starting point the community approach where co‐occurring native and exotic species are compared within the same community (Colautti *et al*., 2004), we hypothesised that, as predicted by the ERH, the abundance of phytophagous nematodes would be less in soils under individuals of *P. elliottii* colonising native shrubby grasslands (locally called *campos de altitude*, henceforth open vegetation) compared with open vegetation without pine (H1). We also hypothesised that *P. elliottii* would exploit the reduction of enemy pressure by root‐feeding nematodes (enemy release) and further reduce their abundance and modify the composition/structure of soil nematode communities in pine plantations (H2a). In alternative to H2a, phytophagous nematodes would increase in pine plantations as generalists become better adapted to the invader and tend to accumulate (H2b; Flory & Clay, 2013; Crous *et al*., 2016; Dickie *et al*., 2017a; Brian & Catford, 2023).

**Fig. 1:**
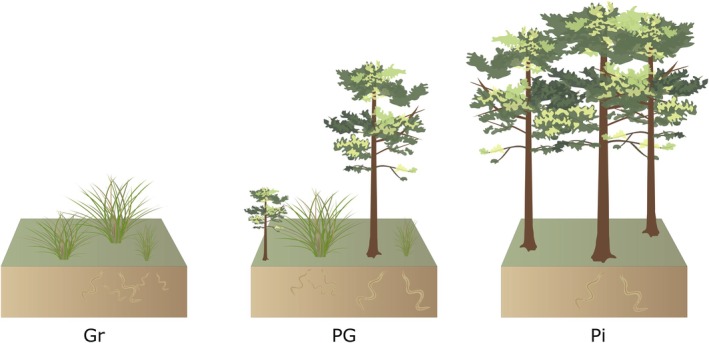
Vegetation types within the Atlantic Forest domain, south‐eastern Brazil. The abundance and functional diversity of soil nematodes was compared among native shrubby grassland locally known as *campos de altitude* and henceforth referred to as open vegetation (Gr; reference point in time, time 0), 20‐ to 30‐yr‐old exotic *Pinus elliottii* individuals invading open vegetation (PG; time 1), and *P. elliottii* plantation (Pi; time 2) established in the late 1950s. Symbols are courtesy of the Integration and Application Network (ian.umces.edu/symbols/), University of Maryland Center for Environmental Science.

To test our hypotheses, we quantified the abundance of phytophagous nematodes in: (1) soils under native open vegetation (control, time 0); (2) under established *P. elliottii* individuals invading open vegetation (time 1; representing up to *c*. 30 yr since establishment); and (3) in a monospecific *P. elliottii* plantation established in the late 1950s, with sparse and species‐poor understorey (time 2; *c*. 65 yr‐old), thus creating, in a space‐for‐time substitution, a virtual chronosequence of invasion (Fig. 1).

## Materials and Methods

### Study area

The study was carried out at a tropical montane forest site (22°39′S and 45°26′W–45°30′W; 1550 m above sea level), in a conservation area in the state of São Paulo, Brazil. The climate is humid subtropical – oceanic characterised by warm and wet summers and temperate dry winters (Cfb by Köppen classification), with no negative hydrological balance. The average annual temperature between 1973 and 2000 was 14.7°C, and the mean annual rainfall was 1813 mm, with most precipitation falling between October and March.

The soils (latosols, podzols and entisols) have formed over metamorphic rocks with various types of orthogneiss (e.g. gneissified granite, gneiss, migmatite, aplite, granulite; Seibert *et al*., 1975; Modenesi‐Gauttieri & Hiruma, 2004). They vary in texture and thickness and are characterised by a low pH, low cation exchange capacity and base saturation and high values of exchangeable aluminium (Oliveira *et al*., 1975). The vegetation consists of a mosaic of secondary forest patches in various stages of regeneration, open vegetation and plantations of exotic conifers and of eucalyptus (*c*. 20%). The native forest is characterised by two native conifers, *Araucaria angustifolia* (Bertol.) Kuntze (an endemic species threatened of extinction; IUCN, 2011) and *Podocarpus lambertii* Klotzsch ex Endl., and various angiosperms, with the emergent layer being dominated by *A. angustifolia*. Open vegetation is characterised by highly diverse herbaceous vegetation, shrubs and scattered small trees (Seibert *et al*., 1975).

The site history includes livestock husbandry, subsistence farming and timber extraction over *c*. 150 yr, up to the late 1970s. The Forestry Institute of São Paulo undertook a reforestation programme of deforested areas with exotic conifers in the late 1950s and early 1960s (Anonymous, 2015). Some of the species, such as *P. elliottii*, *P. taeda* or *P. patula* have become invasive and have been colonising open vegetation.

*Pinus elliottii* is a species native to North America and is among the known invasive species in the Southern Hemisphere (Simberloff *et al*., 2010). Species of the genus *Pinus* are generally tolerant of dry conditions, nutrient‐poor soils and fire regimes (Zanchetta *et al*., 2007). *Pinus elliottii* has been reported to invade savanna (de Abreu & Durigan, 2011; Brewer *et al*., 2018) as well as coastal sandy plains (Bechara *et al*., 2013), open *A. angustifolia* secondary forests (Zenni & Simberloff, 2013) and open montane vegetation (Milani *et al*., 2020), such as that at our long‐term ecological research site in the Mantiqueira range, south‐eastern Brazil. It produces a large number of winged seeds (2–3.6 M seeds ha^−1^yr^−1^; Bechara *et al*., 2013; Miashike, 2015) that are efficiently dispersed by wind (de Abreu & Durigan, 2011). The density of individuals in invaded Brazilian savanna can reach *c*. 3500 individuals ha^−1^ (de Abreu & Durigan, 2011) with pulses of invasion (5–7 yr) likely to be linked to founder trees reaching maturity and to favourable climatic events (Brandes *et al*., 2020). Pine canopy cover/density for similarly aged pine stands has been found to be significantly greater in the introduced (Brazilian savanna) than in the native range (savannas in Mississippi; Brewer *et al*., 2018).

### Sampling design

Soil samples were collected under open native vegetation (control, time 0), under 20‐ to 30‐yr‐old pine individuals invading native open vegetation (time 1), and under pine individuals in a pine plantation established in 1958–1959 *c*. 65 yr ago, thinned twice (last time in 1984), with the removal of 30% of standing timber volume, followed by natural regeneration (São Paulo State Forest Institute archives, w/o date; time 2; Fig. 1, Supporting Information Fig. S1). This allowed us to quantify the relationship between time since invasive pine establishment and the abundance and diversity of phytophagous nematodes.

Eight sample points were randomly selected in the plantation using the ‘Sample’ module in TerrSet (https://clarklabs.org/terrset/). The selection of target trees in the open vegetation, was determined by their available position. Within the open vegetation, we sampled soil under invasive pines and under native species not affected by the presence of pine. A minimum distance of 50‐m between samples (in both open vegetation and plantation) was observed to avoid spatial autocorrelation and guarantee the independence between samples (Buscardo *et al*., 2018).

The soil samples were collected in the austral winter (August 2019) using a soil corer with an internal diameter of 5.7 cm, equipped with a plastic liner tube (Giddings Machine Co., Fort Collins, CO, USA). For each sampling point, four soil cores, separated by at least 1.5 m, were collected around a target tree in the plantation and in invaded open vegetation and around a reference point under native species not affected by the presence of pine. The mineral layer (top 10 cm) and the organic horizon (lacking in open vegetation) were separated and the samples per sampling point were bulked to form composite samples. A total of 40 composite soil samples (16 organic horizon, 24 mineral layer) were collected and stored in a cool box for transporting within 12–24 h to the laboratory, where they were refrigerated at 4°C before processing.

### Extraction of nematodes

The samples were sieved and homogenised using a 2‐mm sieve. One hundred grams of the homogenised soil were used for the nematode extraction with the centrifuge flotation method with sugar (EPPO, 2013). The method allows to isolate nematodes from soil by leveraging their lower density to separate them from heavier soil particles. Soil samples were washed and sieved and then centrifuged in a sugar solution. The suspension containing the nematodes was collected in Erlenmeyer flasks and gradually heated to 60°C and subsequently fixed in formaldehyde (0.27 ml of formaldehyde per 10 ml of suspension). The vials containing the suspension of nematodes were left to rest for 24 h, reduced to 10 ml by siphoning off the extra liquid and kept refrigerated at 4°C for subsequent quantification and identification of nematodes.

### Nematode quantification and identification

The quantification of nematodes was made by using Peter's microscope slides and an Olympus CL2 light microscope (×100 magnification). Each sample was manually stirred, and an aliquot of 2 ml of the extraction (20% of the total) was taken for quantification. The nematodes on the slide were counted and the number obtained was multiplied by five to estimate the total abundance per sample. Photographic records were taken by using Olympus Cellsens.

The identification of nematodes was carried out in the Department of Phytopathology and Nematology, School of Agriculture Luiz de Queiroz, University of São Paulo, Brazil. The specimens collected from the Peter's slides were mounted on semi‐permanent microscope slides with 2% formaldehyde for identification under a light microscope (Leica DMLB at ×1000 magnification). Of the eight samples extracted five were randomly drawn for each of the three vegetation types, obtaining a total of 15 samples, which were processed for identifying trophic groups (Yeates *et al*., 1993). Morphological attributes of the buccal region were used for the taxonomic identification of the specimens to the genus level (Coleman & Wall, 2007). Nematode taxonomic richness and Shannon‐Wiener and Simpson diversity indices at the genus level were quantified for each vegetation type. Abundances were expressed as number of individuals per 100 g soil dry weight.

### Data analyses

Since the open vegetation lacked almost completely the organic layer, we focussed the analyses on the mineral layer samples. Differences among vegetation types in total nematode abundance, richness and diversity were determined by carrying out one‐way analyses of variance (ANOVA), while differences in abundance among nematode trophic groups per vegetation type were determined by two‐way ANOVA after testing for normality of the data and homogeneity of the variances. Data were square‐root‐transformed to meet the assumptions of ANOVA. Differences among groups were evaluated with the Tukey test. Differences in nematode community structure among vegetation types were visualised by NMDSs (vegan R package; Oksanen *et al*., 2013) and assessed both on presence/absence and abundance data of nematode genera with the ADONIS function (vegan) with 10 000 permutations.

The relative abundance of trophic groups in the three vegetation types, and the total number of taxa and the taxa of each trophic group shared among, or unique to each vegetation type were calculated (venndiagram R package; Chen & Boutros, 2011). The distribution of all nematode genera and that of phytophagous genera among vegetation types were analysed using the function ecospatC‐score with 10 000 permutations in the ecospat R package (Di Cola *et al*., 2017; Broennimann, 2018). The function tests by using a standard null model approach (Gotelli, 2000) for non‐random patterns of co‐occurrence of taxa, that is, whether or not genera co‐occur more or less than expected by chance.

Data manipulation and statistical analyses were carried out using tidyverse (Wickham & Wickham, 2017), ggpubr (Kassambara, 2018), rstatix (Kassambara, 2019), dplyr (Wickham *et al*., 2020), emmeans (Lenth, 2022) and biodiversityr (Kindt & Coe, 2005) R packages.

## Results

### Total nematode abundance

**Fig. 2:**
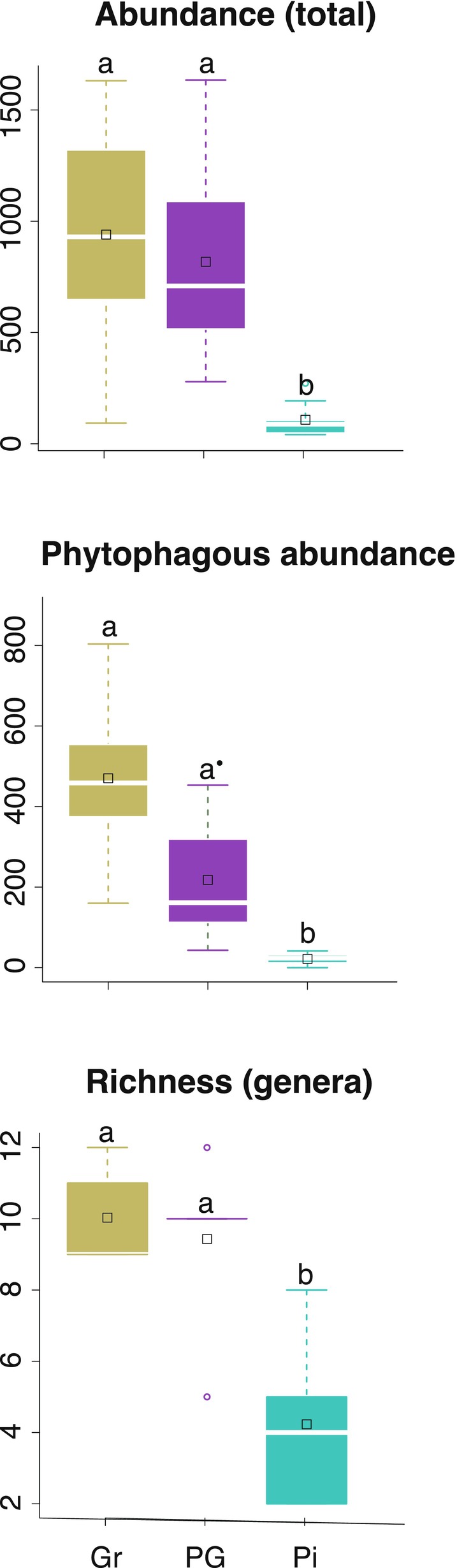
Abundance of total nematodes and of phytophagous nematodes in soil (100 g dry weight) and nematode richness at the genus level under native vs. exotic vegetation in montane ecosystems of the Atlantic Forest domain, south‐eastern Brazil. Gr, native open vegetation locally known as *campos de altitude*; PG, individuals of exotic *Pinus elliottii* invading native open vegetation; Pi, *P. elliottii* plantation. Significant differences among vegetation types (one‐way ANOVA) are indicated by different letters (see Supporting Information Tables S1, S2, S4). Boxplots: centre line, median; empty square, mean value; box limits, 25^th^ and 75^th^ percentiles; whiskers, 1.5 times interquartile range; empty circles, outliers; •, *P* = 0.092.

Overall mean ± SE total nematode abundance per 100 g dry soil was 557 ± 102. For the open vegetation, it was 835 ± 188, for *P. elliottii* individuals in the open vegetation, it was 727 ± 165 and for the *P. elliottii* plantation, it was 108 ± 26 (Fig. 2). Total nematode abundance was significantly lower in the *P. elliottii* plantation than under open vegetation or under pines established in the open vegetation (ANOVA, *F*_(2,24)_ = 6.56; *P* = 0.005; Tables S1, S2; Dataset S1).

### Nematode richness and diversity

A total of 49 genera were identified (26 in the open vegetation; 28 under *P. elliottii* colonising open vegetation; 17 in the *P. elliottii* plantation; Table 1; Dataset S1). Some genera, such as *Acrobeles*, *Aphelenchoides*, *Helicotylenchus*, *Tylenchus*, and *Xiphinema* were ubiquitous, whereas others were found exclusively in a single vegetation type. Some plant‐feeding taxa, including the genera *Hemicycliophora*, *Meloidogyne*, *Discocriconemella*, and *Rotylenchus*, were found in native vegetation but not in the soil in the *P. elliottii* plantation or under *P. elliottii* in the open vegetation.

**Table 1:** Taxonomic identity of nematodes extracted from mineral topsoil (0–10 cm)under native and exotic vegetation in montane ecosystems of the Atlantic Forest domain, south‐eastern Brazil.

Family	Genus	Trophic group	Gr	PG	Pi
Alloionematidae	*Rhabditophanes*	3	−	+	−
Anguinidae	*Nothotylenchus*	2	+	−	−
Aphelenchidae	*Aphelenchus*	2	−	−	+
Aphelenchoididae	*Aphelenchoides*	2	+	+	+
Aphelenchoididae	*Seinura*	5	−	+	−
Aulolaimoididae	*Aulolaimoides*	3	−	+	−
Belondiridae	*Opailaimus*	8	+	−	−
Belonolaimidae	*Tylenchorhynchus*	1	−	−	+
Cephalobidae	*Acrobeles*	3	+	+	+
Cephalobidae	*Acrobeloides*	3	−	+	+
Cephalobidae	*Cephalobus*	3	+	−	−
Cephalobidae	*Cervidellus*	3	−	+	−
Cephalobidae	*Eucephalobus*	3	+	−	+
Criconematidae	*Criconema*	1	+	+	−
Criconematidae	*Discocriconemella*	1	+	−	−
Criconematidae	*Hemicriconemoides*	1	−	−	+
Criconematidae	*Macrophostonia*	1	−	+	−
Criconematidae	*Mesocriconema*	1	−	+	+
Diplogastridae	*Diplogaster*	3	−	+	−
Diploscapteridae	*Diploscapter*	3	+	+	−
Dorylaimidae	*Eudorylaimus*	8	−	+	+
Hemicycliophoridae	*Hemicycliophora*	1	+	−	−
Hoplolaimidae	*Helicotylenchus*	1	+	+	+
Hoplolaimidae	*Rotylenchus*	1	+	−	−
Leptonchidae	*Utahnema*	8	+	−	−
Longidoridae	*Xiphidorus*	1	−	−	+
Longidoridae	*Xiphinema*	1	+	+	+
Meloidogynidae	*Meloidogyne*	1	+	−	−
Mononchidae	*Mononchus*	5	+	−	−
Mononchidae	*Prionchulus*	5	+	−	−
Mylonchulidae	*Sporonchulus*	5	+	−	−
Odontopharyngidae	*Odontopharynx*	3	−	+	−
Panagrolaimidae	*Panagrolaimus*	3	−	+	−
Panagrolaimidae	*Turbatrix*	3	+	−	−
Paratylenchidae	*Gracilacus*	1	−	+	−
Paratylenchidae	*Paratylenchus*	1	+	+	−
Plectidae	*Wilsonema*	3	−	+	−
Pratylenchidae	*Pratylenchus*	1	−	+	−
Prismatolaimidae	*Prismatolaimus*	3	+	−	+
Rhabditidae	*Rhabditis*	3	+	+	−
Rhabditidae	*Teratorhabditis*	3	−	+	−
Teratocephalidae	*Teratocephalus*	3	−	−	+
Thornenematidae	*Thornenema*	8	+	+	−
Trichodoridae	*Paratrichodorus*	1	−	+	−
Tylenchidae	*Ecphyadophora*	1	+	−	+
Tylenchidae	*Tylenchus*	2	+	+	+
Tylencholaimellidae	*Tylencholaimellus*	2	−	−	+
Tylencholaimidae	*Meylonema*	2	−	+	−
Tylencholaimidae	*Tylencholaimus*	2	+	+	−

Trophic groups are according to Yeates *et al*. (1993; 1, phytophage; 2, mycophage; 3, bacteriophage; 5, predator, 8, omnivore); presence (+) or absence (−) of identified genera; Gr, native open vegetation locally known as *campos de altitude*; PG, individuals of exotic *Pinus elliottii* invading native open vegetation; Pi, *P. elliottii* plantation.

**Fig. 3:**
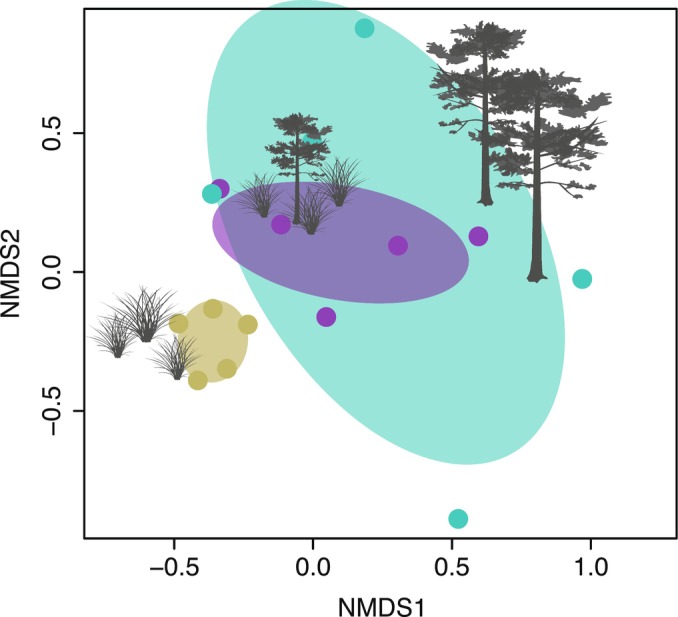
Non‐metric multidimensional scaling (NMDS) ordination plot based on abundance data of soil nematode communities (genus level) in native open vegetation and under exotic *Pinus elliottii* in montane ecosystems of the Atlantic Forest domain, south‐eastern Brazil. Stress 3D, 0.138; sand colour, native open vegetation; purple, individuals of *P. elliottii* invading native open vegetation; turquoise, *P. elliottii* plantation. Ellipses denote 95% confidence intervals using the SE of the weighted average sample scores per vegetation type. Symbols are courtesy of the Integration and Application Network (ian.umces.edu/symbols/), University of Maryland Center for Environmental Science.

Soil in the *P. elliottii* plantation had significantly lower values of nematode taxonomic richness than the other vegetation types (ANOVA, *F*_2,12_ = 10.17; *P* = 0.003; Fig. 2). No differences among vegetation types were found, when comparing either the Shannon diversity (ANOVA, *F*_2,12_ = 0.44; *P* = 0.657; Fig. S2; Table S1), or the Simpson dominance index values (ANOVA, *F*_2,12_ = 0.86; *P* = 0.448; Fig. S2; Table S1). The nematode communities in the native vegetation were distinctly different from those under pine individuals invading open vegetation (presence/absence: *F* = 3.4, *R* ^2^ = 0.30, *P* = 0.008; abundance: *F* = 2.6, *R* ^2^ = 0.24, *P* = 0.017) and from those in the pine plantation (presence/absence: *F* = 2.7, *R* ^2^ = 0.25, *P* = 0.008; abundance: *F* = 2.9, *R* ^2^ = 0.27, *P* = 0.008; Figs 3, S3). Nematode communities under pine individuals invading open grasslands differed from those in the pine plantation when considering abundance data (abundance: *F* = 1.6, *R* ^2^ = 0.17, *P* = 0.033; presence/absence: *F* = 1.4, *R* ^2^ = 0.15, *P* = 0.164; Figs 3, S3).

The most abundant and frequent nematode taxa were reduced in numbers or absent under pine. We recorded an eightfold decrease in nematode abundance and 2.5‐fold decrease in taxonomic richness in the pine plantation compared with those in native open vegetation. Frequently occurring taxa (i.e. those in > 80% of samples) were reduced by 60% under pine invading open vegetation and were absent altogether in the pine plantation. Samples collected under the pine plantation were much dispersed in the ordination plot, especially compared to those of the open vegetation, because communities were less structured and this contributed to the increased distance among samples in the pine plantation. This was more evident when abundance data were considered (Figs 3, S3).

Open vegetation and pine individuals invading open vegetation shared 11 genera, whereas open vegetation and pine plantation and pine plantation and pine individuals invading open vegetation shared eight genera. There were 12 genera unique to open vegetation, 14 to pine individuals invading open vegetation and 6 to pine plantation (Fig. S4). Phytophagous nematodes were represented by nine genera in open vegetation and under pine individuals invading open vegetation and by seven in pine plantation. There were four genera shared between open vegetation and pine individuals invading open vegetation and three between open vegetation and pine plantation as well as between pine individuals invading open vegetation and pine plantation (Fig. S4). There were four phytophagous genera unique to open vegetation and pine individuals invading open vegetation, while pine plantation had three unique phytophagous genera (Fig. S4).

### Nematode trophic groups

**Fig. 4:**
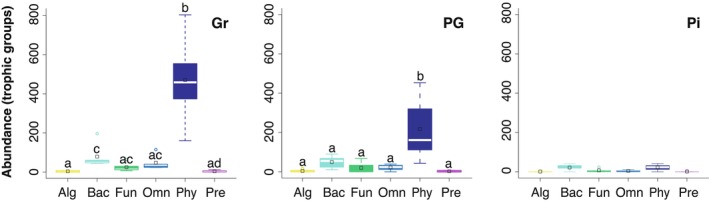
Abundance of nematode trophic groups in soil (100 g dry weight) under native vs. exotic vegetation in montane ecosystems of the Atlantic Forest domain, south‐eastern Brazil. Gr, native open vegetation; PG, individuals of exotic *Pinus elliottii* invading native open vegetation; Pi, *P. elliottii* plantation; Alg, algivore; Bac, bacterivore; Fun, fungivore; Omn, omnivore; Phy, phytophage; Pre, predator. Significant differences among trophic groups (two‐way ANOVA) are indicated by different letters (see Supporting Information Tables S1, S3). Boxplots: centre line, median; empty square, mean value; box limits, 25^th^ and 75^th^ percentiles; whiskers, 1.5 times interquartile range; empty circles, outliers.

Phytophagous nematodes represented the most abundant group in all vegetation types (ANOVA, *F*_2,72_ = 37,42; *P* ≤ 0.001; Tables S1, S3), except in the *P. elliottii* plantation where there were no significant differences among trophic groups (Fig. 4).

Open vegetation had a significantly larger abundance of phytophagous nematodes (470 ± 106 per 100 g dry soil) than the pine plantation (22 ± 7) and soil under *P. elliottii* in the open vegetation (218 ± 74; ANOVA, *F*_5,72_ = 24.08; *P* ≤ 0.001; Fig. 2; Tables S1, S4). Soil under native open vegetation and *P. elliottii* in the open vegetation had significantly larger abundance of phytophagous nematodes when compared to that in the pine plantation (Fig. 2; Table S4). Significant differences among vegetation types were also observed for bacterivores and omnivores; both groups showed lower abundance in pine plantation than in the native open vegetation (Table S4).

The relative abundance of phytophagous nematodes decreased when comparing open vegetation (74.7%) with pine invading open vegetation (68.9%) and with pine plantation (38%; Fig. S5). Other trophic groups showed either increasing values or no changes in their relative abundances from open vegetation to pine invading open vegetation and plantation. The groups that contributed most to these changes were bacterivores (open vegetation, 12.5%; pine invading open vegetation, 15.7%; pine plantation, 37.9%) and fungivores (open vegetation, 3.9%; pine invading open vegetation, 6.4%; pine plantation, 12.4%; Fig. S5). Neither the distribution of all genera considered together nor that of phytophagous nematode genera deviated from random expectations within vegetation types. Standardised effect size ([observed value – mean of the null distribution]/standard deviation of the null distribution) values ranged between −1.25 and 1.38, that is, ≤ |2|, indicating neither spatial aggregation nor segregation, that is, random pattern of taxa co‐occurrence (see Table S5).

## Discussion

Our study highlights that *P. elliottii*, an exotic pine species introduced in the Southern Hemisphere from North America that has been invading open vegetation in the Atlantic Forest and in other biogeographic domains, is associated with a reduced root‐feeding soil nematode abundance compared with that in native open vegetation. Phytophagous nematodes were the most abundant trophic group in all vegetation types, but decreasingly less so under pine individuals invading open vegetation (time 1) and remarkably depauperate in the pine plantation established in the late 1950s (time 2) compared with that in native open vegetation (time 0). The results lead us to suggest that *P. elliottii* might have escaped from phytophagous pressure in its new range and by modifying soil attributes and nematode communities further reduced the abundance of phytophagous nematodes in its rhizosphere (Reinhart & Callaway, 2006). The impact of *P. elliottii* on nematode communities was mainly driven by changes in the trophic structure with a drastic depletion of abundant and frequent phytophagous species and overall nematode abundance and richness in the pine plantation.

Our community approach supported one of the premises of the ERH, whereby we found a lower load of phytophagous nematodes under pine individuals in open vegetation than under native vegetation not impacted by pine (H1). The abundance of phytophagous nematodes in the pine plantation decreased twofold compared with that in native open vegetation. In terms of the ERH, the lower abundance of phytophagous nematodes in soils under pine individuals in open vegetation than under native vegetation not invaded by pine could be partly explained by highly species‐specific interactions between nematodes and host plants and/or by the lack of co‐introduced phytophagous nematode species with pine from its native range. When exotic species are not phylogenetically closely related with co‐occurring native species they are likely to experience lower specialist diversity and lower impacts from generalists, as enemies in the invaded range are less adapted to target them (Brian & Catford, 2023). Native vegetation at our study site is generally colonised by arbuscular mycorrhizal fungi and is characterised by a highly diverse and endemic‐rich herbaceous vegetation, shrubs and scattered small trees which are phylogenetically very distant from *P. elliottii* (Robim & Pfeifer, 1988; Dornelles *et al*., 2021). Processes of coevolution between specialist nematodes and host plants in these natural ecosystems might therefore have been contributing to plant species‐specificity, resulting in lower phytophagous nematode associations with exotic pines. Additionally, the survival of specialist phytophagous nematodes could be compromised in the absence of the host (Perry & Moens, 2011) which could have led to low abundances of native nematodes under the exotic species. This could have been the case at our study site, where native vegetation cover is progressively suppressed as pine individuals invading open grasslands increase their canopy cover. The lower number of phytophagous nematodes observed in the plantation than under pine individuals invading open grasslands suggests also that specialist enemies were either not co‐introduced with pine or that they did not overcome some biotic and/or environmental filters, conversely to what has been observed for some ECM fungal species that were co‐introduced with pine and became co‐invasive.

Our hypothesis (H2a) that *P. elliottii* could benefit from the initial reduction of the enemy pressure represented by root‐feeding nematodes in its exotic range and then further reduce their abundance and modifying the composition/structure of soil nematode communities, was confirmed by our results. While the relationship between *P. elliottii* and the abundance of phytophagous nematodes was related to the stage of invasion of natural ecosystems, the abundance of root feeding nematodes was significantly lower (10‐fold decrease) in the pine plantations (closed canopy pine cover sustained for over 65 yr) than under scattered pine individuals invading open vegetation. This contradicts our alternative hypothesis (H2b) that invasive plants accumulate enemies after establishment (either via co‐invasion of non‐native enemies and/or via adaptation or acclimation of resident enemies; Flory & Clay, 2013; Crous *et al*., 2016; Dickie *et al*., 2017b).

Community composition was significantly different among vegetation types and was mainly related to differences in nematode trophic structure, with a major depletion of abundant/frequent phytophagous taxa and overall nematode abundance/richness in the pine plantation. The distributions of nematode communities/phytophagous nematodes within vegetation types did not deviate from random expectations suggesting that the nematode communities, even if significantly different from one another, are mainly structured by stochastic processes. A random pattern of species co‐occurrence has been found in previous studies suggesting that neutral processes are the main drivers of the assembly of nematode communities at the landscape scale and could be attributable to dispersal limitation and soil patchiness (Ettema *et al*., 2000; Zinger *et al*., 2019). While some soil microorganisms may be dispersed by wind, the dispersal of larger‐sized soil biota requires active movement, and the dispersal capacity/distance of nematodes is estimated to range from 0.1 to 1 m yr^−1^ (Bardgett & van der Putten, 2014). Being dispersal limited, nematodes are therefore susceptible to environmental heterogeneity at the local scale and related spatial processes, that is, being deterministically associated with environmental heterogeneity, which itself can be rather stochastic.

Nematode communities may be affected by the presence of pine in multiple and nonexclusive ways. The negative relationship between the abundance of phytophagous nematodes and the stage of pine invasion of native vegetation at our study site suggest that *P. elliottii* might benefit from the reduction of the enemy pressure caused by root‐feeding nematodes and further reduce their abundance over time via positive soil biota – plant feedbacks. Previous research comparing nematode communities in soils under native and exotic pine species has found greater nematode abundance/diversity under native plants than under pines and related it to the availability of resources and microhabitat for nematodes, including soil organic matter and microbial biomass (Yeates & Saggar, 1998; Scholes & Nowicki, 2000; Dehlin *et al*., 2008). A major reduction in taxonomic and functional diversity has been reported to have resulted in the biotic homogenisation of soil invertebrate communities in plantations of exotic pines and under colonising individuals, established in invaded native grasslands. The homogenisation was associated with increased mineralisation of nitrogen and depletion of soil carbon (Chapela *et al*., 2001; Dickie *et al*., 2011; Cifuentes‐Croquevielle *et al*., 2020) driven by the inhibition of carbon turnover (Araujo & Austin, 2020). A recent study conducted in the vicinity of our study site has quantified soil microbial biomass, soil mineral nitrogen and functional genes involved in the nitrogen cycle in soils in a *P. taeda* plantation (Muñoz, 2021). The high abundances of the nitrogen fixing *nifH* and ammonia oxidising bacterial and archaeal *amoA* genes found in the plantation have suggested that the pine plantation may have been nitrogen limited and the potential great nitrogen demand of the fast‐growing pine plantation could have led, over time, to soils depleted in nutrients, possibly associated with a decrease in nematode diversity/abundance. These pieces of evidence, albeit indirect, would support the hypothesis that reduced resource availability and/or the introduction of novel litter and root exudates under exotic pine species has a negative impact on nematode abundance and diversity.

The abundance of phytophagous nematodes could also be reduced by the presence of defence compounds produced *by P. elliottii* (‘novel weapon’ hypothesis; Callaway & Ridenour, 2004). Pine species exert different defence responses against the nematode *Bursaphelenchus xylophilus* (Steiner & Bührer) Nickle in the xylem including phytohormone signalling, secondary metabolism (e.g. terpenoids, phenylpropanoids), oxidative stress, plant defence response and resistance (Modesto *et al*., 2022). Similarly, one may speculate in the absence of evidence that pine trees might produce defence products against root‐feeding nematodes akin to that reported by Modesto *et al*. and which might not be produced by native species.

The alteration of native plant communities by exotic pine species is accompanied by changes in belowground diversity (Sapsford *et al*., 2022) and these processes are likely to affect biogeochemical cycles and ecosystem functioning. The fact that nematode communities were least structured under the pine plantation further supports the positive feedback hypothesis by putting in evidence the disruption of nematode communities by *P. elliottii*. The reduced taxonomic and functional belowground diversity in ecosystems dominated by exotic pine is likely to reduce the number of trophic interactions both aboveground and belowground, with losses of diversity and multifunctionality (Keith *et al*., 2009; Zhang *et al*., 2019).

Our study has two notable limitations. First, nematodes were assessed exclusively in the austral winter, which coincides with low precipitation when compared with the wet summer in south‐eastern Brazil. Seasonality has previously been observed to affect the composition and structure of nematode communities (Girgan *et al*., 2020) as well as nematode resources including bacteria and fungi (Buscardo *et al*., 2018, 2024). By taking a one‐time snapshot, we were unable to morphologically identify most juvenile individuals and thus it may be that our results underestimate nematode richness and abundance within trophic groups. Second, nematodes were isolated exclusively from the mineral horizon (the organic layer was almost absent in the open vegetation without pine), and it is therefore likely that overall nematode abundance was underestimated. Nonetheless, our mean total nematode abundance data (4.18 M m^−2^) falls within the range reported by Yeates (2007) for *Pinus* species (3.25 M to 6.95 M m^−2^).

In conclusion, our study shows that over time, established pine in its exotic range in the Atlantic forest domain, is associated with a low abundance of root‐feeding nematodes, which is likely to be related to: an initial reduced enemy pressure on pine in the early stages of establishment and supporting therefore the premises of the ERH; and a change towards potential positive plant–soil feedbacks in the longer‐term. The findings suggest that both enemy release from phytophagous nematodes and plant–soil feedbacks are likely to contribute, together with pine dispersal capacity and morphological/physiological traits, to the invasiveness of pine species in montane ecosystems in the Southern Hemisphere. Nonetheless, a deeper understanding of plant–soil interactions in response to exotic plant populations to novel belowground communities in the introduced range is needed to be able to project exotic species invasions shaped by interacting global change drivers and their implications for ecosystem processes.

## Competing interests

None declared.

## Author contributions

LN and EB planned the research; LN and LSCG undertook the fieldwork; LSCG extracted and counted soil nematodes; MMI and LSCG identified nematode genera and/or species using the morphology of adult and juvenile forms; LSCG and EB made the statistical analyses; EB led the writing of the manuscript with input from LN, LSCG and MMI. All authors have read and approved the final version of the manuscript.

## Disclaimer

The New Phytologist Foundation remains neutral with regard to jurisdictional claims in maps and in any institutional affiliations.

## Supporting information


**Dataset S1** Taxonomic identity and abundance of nematodes.

**Fig. S1** Sampling area in the Atlantic Forest domain.
**Fig. S2** Nematode diversity.
**Fig. S3** Nonmetric multidimensional scaling ordination plot of soil nematode communities.
**Fig. S4** Nematode genera and trophic groups shared among different vegetation types.
**Fig. S5** Relative abundance of nematode trophic groups in soils under different vegetation types.
**Table S1** Results of ANOVA on soil nematode abundance, richness and diversity and on the abundance of nematode trophic groups among and within different vegetation types.
**Table S2** Results of the Tukey test comparisons of soil nematode abundance and richness among vegetation types.
**Table S3** Results of the Tukey test comparisons of the abundance of different soil nematode trophic groups within different vegetation types.
**Table S4** Results of the Tukey test comparisons of the abundance of soil nematode trophic groups among vegetation types.
**Table S5** Results of a null model analysis to determine the co‐occurrence of total nematode genera and of phytophagous genera among vegetation types.
Please note: Wiley is not responsible for the content or functionality of any Supporting Information supplied by the authors. Any queries (other than missing material) should be directed to the *New Phytologist* Central Office.

## Data Availability

Data supporting the findings of this study are available in Dataset S1.
