# Supplementary material for: Low abundance of phytophagous nematodes under invasive exotic Pinus elliottii – enemy release and plant–soil feedbacks
Source: New Phytol. 2025 Dec 28;249(6):3060–71. doi: 10.1111/nph.70852 (PMC12917447; doi:10.1111/nph.70852)
Supplement: Supplementary file 2 — Fig. S1 Sampling area in the Atlantic Forest domain. Fig. S2 Nematode diversity. Fig. S3 Nonmetric multidimensional scaling ordination plot of soil nematode communities. Fig. S4 Nematode genera and trophic groups shared among different vegetation types. Fig. S5 Relative abundance of nematode trophic groups in soils under different vegetation types. Table S1 Results of ANOVA on soil nematode abundance, richness and diversity and on the abundance of nematode trophic groups among and within different vegetation types. Table S2 Results of the Tukey test comparisons of soil nematode abundance and richness among vegetation types. Table S3 Results of the Tukey test comparisons of the abundance of different soil nematode trophic groups within different vegetation types. Table S4 Results of the Tukey test comparisons of the abundance of soil nematode trophic groups among vegetation types. Table S5 Results of a null model analysis to determine the co‐occurrence of total nematode genera and of phytophagous genera among vegetation types. Please note: Wiley is not responsible for the content or functionality of any Supporting Information supplied by the authors. Any queries (other than missing material) should be directed to the New Phytologist Central Office. [file NPH-249-3060-s002.pdf]

New Phytologist

***New Phytologist* Supporting Information**

Article title: Low abundance of phytophagous nematodes under invasive exotic *Pinus elliottii* – enemy release and plant-soil feedbacks

Authors: Lynda S. C. Guerrero, Erika Buscardo, Mario M. Inomoto, Laszlo Nagy

Article acceptance date: 28 November 2025

The following Supporting Information is available for this article:

Fig. S1 Sampling area in the Atlantic Forest domain

Fig. S2 Nematode diversity

Fig. S3 Non-metric multidimensional scaling ordination plot of soil nematode communities

Fig. S4 Nematode genera and trophic groups shared among different vegetation types

Fig. S5 Relative abundance of nematode trophic groups in soils under different vegetation types

Table S1 Results of ANOVA on soil nematode abundance, richness and diversity and on the abundance of nematode trophic groups among and within different vegetation types

Table S2 Results of the Tukey test comparisons of soil nematode abundance and richness among vegetation types

Table S3 Results of the Tukey test comparisons of the abundance of different soil nematode trophic groups within different vegetation types

Table S4 Results of the Tukey test comparisons of the abundance of soil nematode trophic groups among vegetation types

Table S5 Results of a null model analysis to determine the co-occurrence of total nematode genera and of phytophagous genera among vegetation types

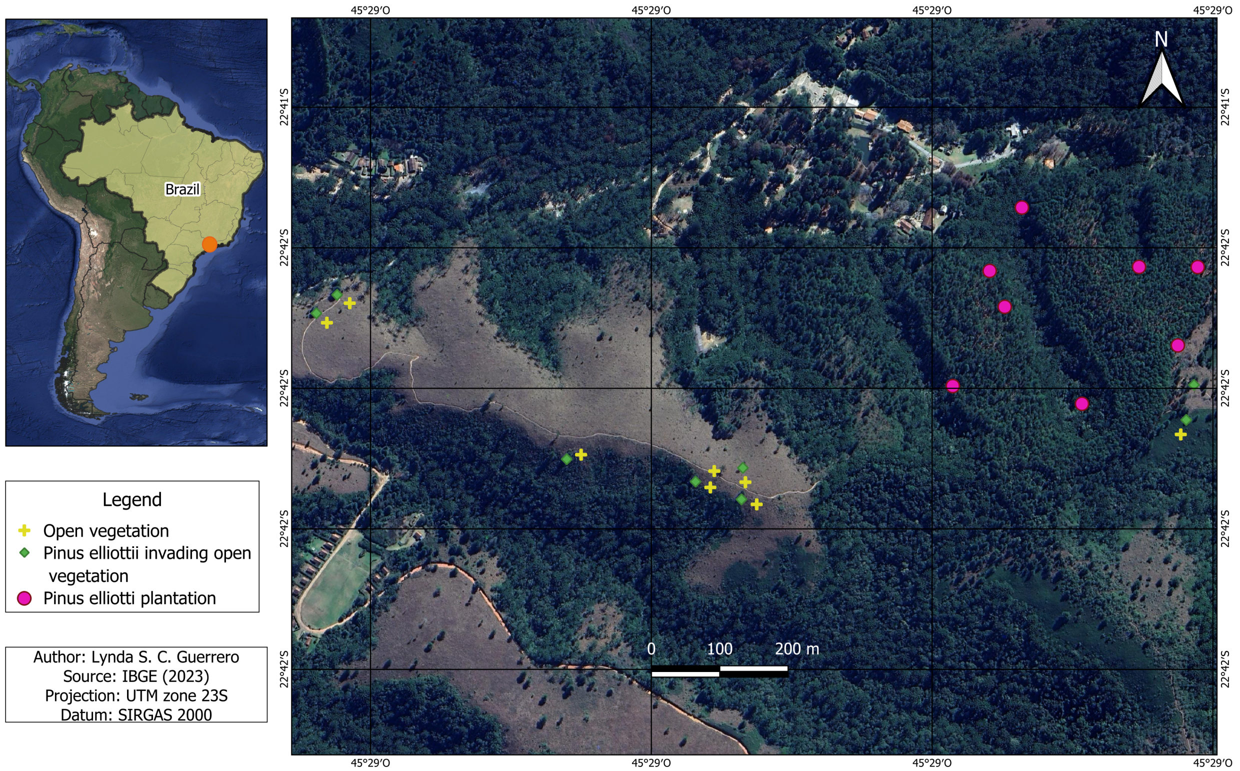

Fig. S1 Sampling area in the Atlantic Forest domain in the State Park of Campos do Jordão, São Paulo state, Brazil. Soil samples for nematode extraction were collected in the austral winter (August 2019) at eight random points per vegetation type (i.e. native open vegetation locally known as campos de altitude), exotic *Pinus elliottii* invading open vegetation, and *P. elliottii* plantation). The random point coordinates were generated using the 'Sample' module in TerrSet (https://clarklabs.org/terrset/). The sampling points were separated by at least 50 m to avoid spatial autocorrelation. The soil samples were collected with a soil corer with an internal diameter of 5.7 cm, using a plastic liner tube (Giddings Machine Company, Fort Collins, CO, USA). For each sampling point, four soil cores, separated by at least 1.5 m, were collected. The organic (variable thickness) and mineral soil (top 10 cm) layers were separated, and the mineral layer was bulked to form composite samples per sampling point. The map was created using Q-GIS program, version 3.40.0.

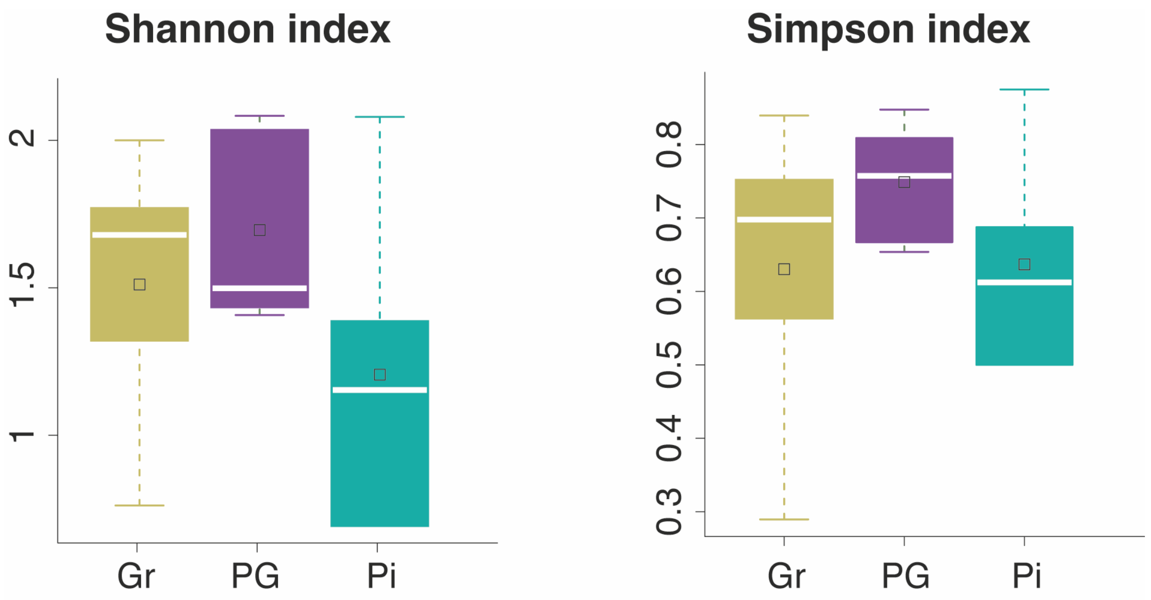

Fig. S2 Nematode diversity (genera) in soils (100 g dry weight) under native versus exotic vegetation in montane ecosystems of the Atlantic Forest domain, south-eastern Brazil. Differences among vegetation types were tested with one-way ANOVA (see Table S1, S2). Gr, native open vegetation; PG, individuals of exotic *Pinus elliottii* invading native open vegetation; Pi, *P. elliottii* plantation. Boxplots: centre line, median; empty square, mean value; box limits, 25th and 75th percentiles; whiskers, 1.5 times interquartile range; empty circles, outliers.

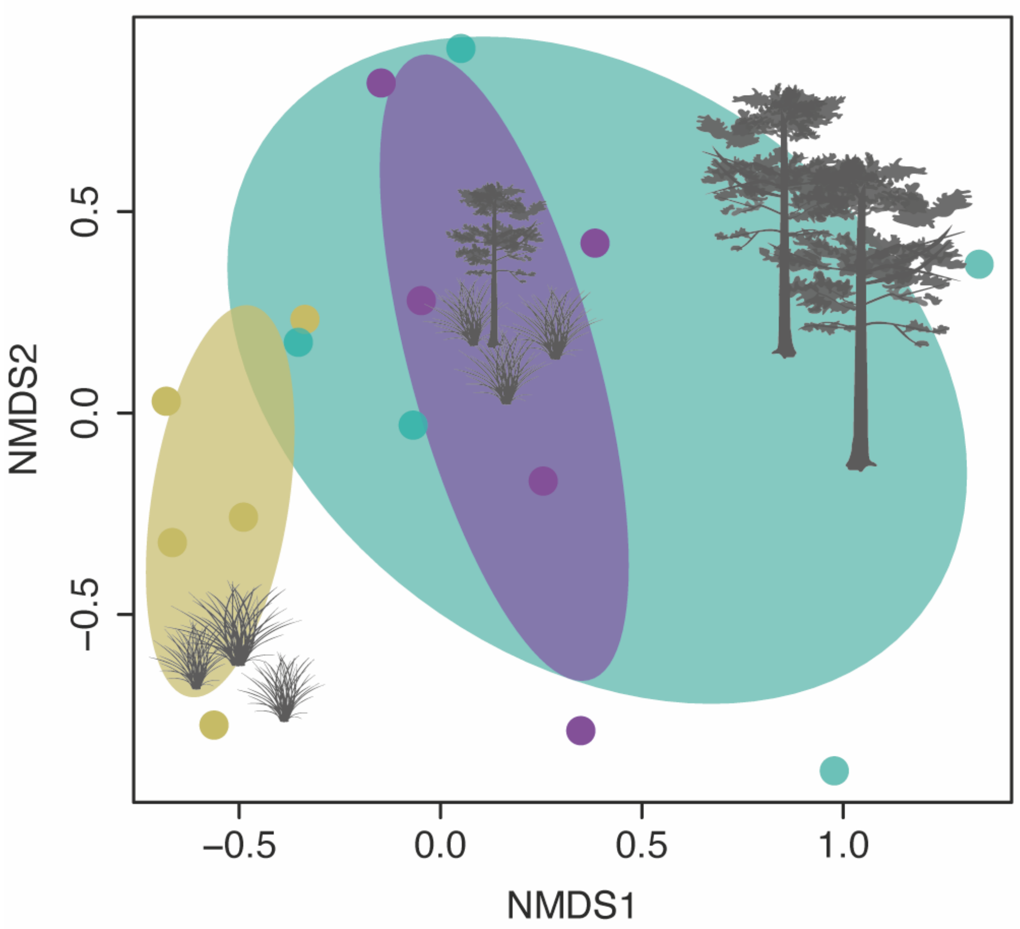

Fig. S3 Non-metric multidimensional scaling (NMDS) ordination plot based on presence / absence data of soil nematode communities (genus level) in native open vegetation and under the exotic *Pinus elliottii* in montane ecosystems of the Atlantic Forest domain, south-eastern Brazil. Stress 3D, 0.138. Sand colour, native open vegetation; purple, established individuals of exotic *Pinus elliottii* invading native open vegetation; turquoise, *P. elliottii* plantation. Ellipses denote 95% confidence intervals using standard error of the weighted average sample scores per vegetation type. Symbols are courtesy of the Integration and Application Network (ian.umces.edu/symbols/), University of Maryland Center for Environmental Science.

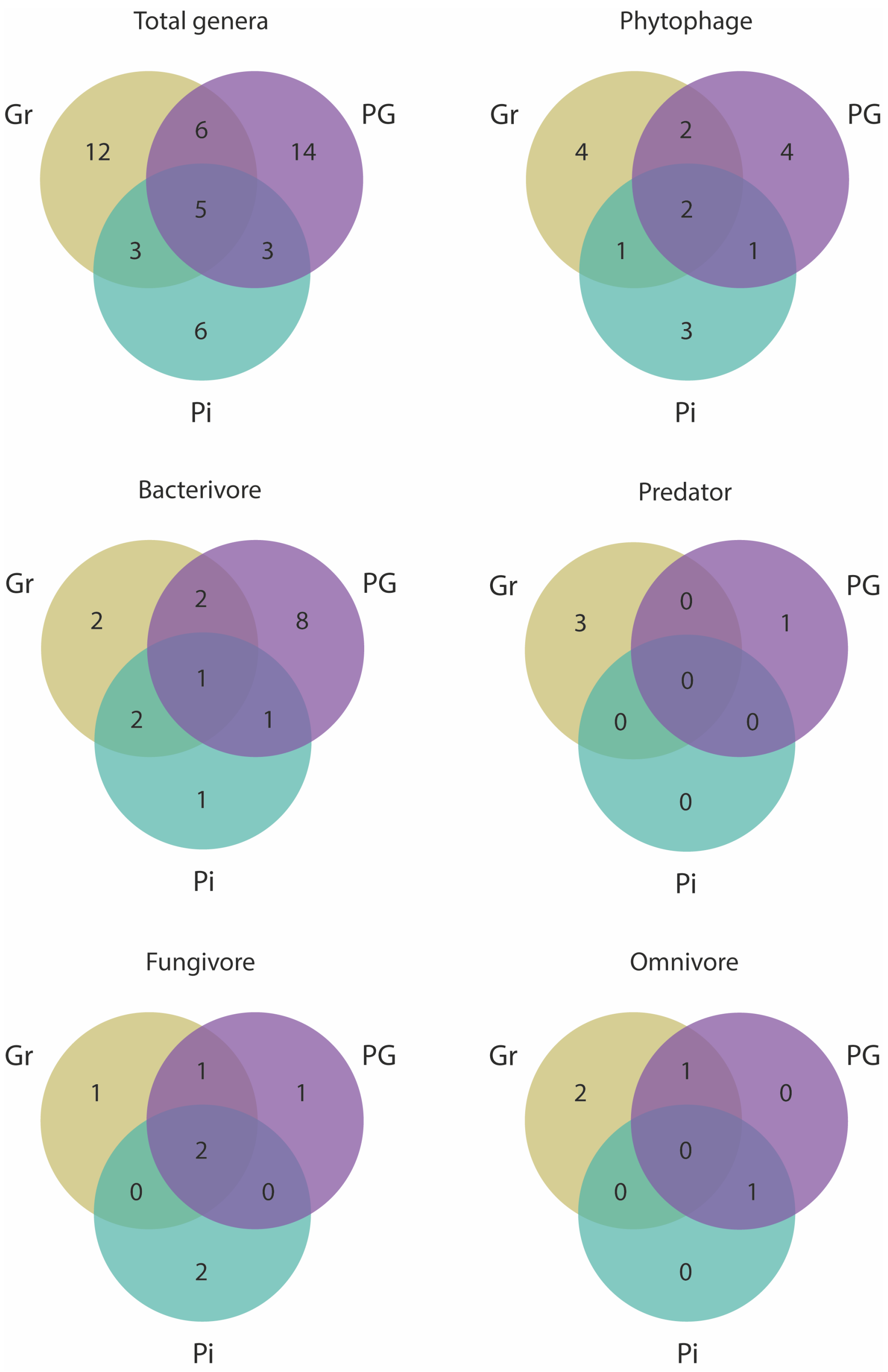

Fig. S4 Venn diagrams showing the observed total number of soil nematode genera and trophic groups shared between native open vegetation and the exotic *Pinus elliottii* in montane ecosystems of the Atlantic Forest domain, south-eastern Brazil. Gr, native open vegetation; PG, individuals of exotic *Pinus elliottii* invading native open vegetation; Pi, P*. elliottii* plantation.

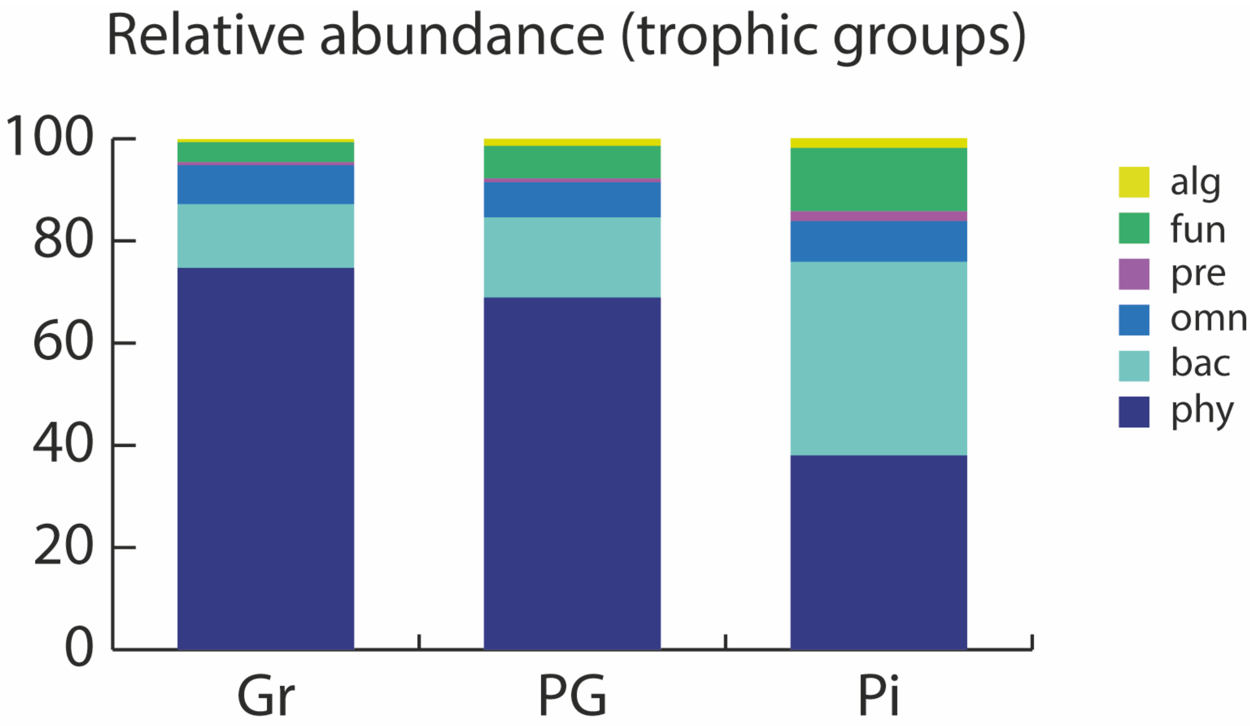

Fig. S5 Relative abundance of nematode trophic groups (including those which were not identified at the genus level) in soils under native open vegetation and exotic pine in montane ecosystems of the Atlantic Forest domain, south-eastern Brazil. Gr, native open vegetation; PG, individuals of exotic *Pinus elliottii* invading native open vegetation; Pi, *P. elliottii* plantation; Alg, algivore; Bac, bacterivore; Fun, fungivore; Omn, omnivore; Phy, phytophage; Pre, predator.

Table S1 Results of analyses of variance (ANOVA) on (a) soil nematode abundance (total, one-way ANOVA) and (b) nematode richness and diversity (genera, one-way ANOVA) among native open vegetation, open vegetation invaded by individuals of exotic *Pinus elliottii*, and a plantation of *P. elliottii*; and on (c) the abundance of nematode trophic groups (two-way ANOVA) between and within vegetation types in montane ecosystems of the Atlantic Forest domain, south-eastern Brazil.

(a)

	**DFn**	**DFd**	***F***	***P***	**ges**
**Abundance (total)**	2	24	6.562	0.005	0.354

(b)

	**DFn**	**DFd**	***F***	***P***	**ges**
**Richness**	2	12	10.17	0.003	0.629
**Shannon index**	2	12	0.435	0.657	0.068
**Simpson index**	2	12	0.86	0.448	0.125

(c)

**Effect**	**DFn**	**DFd**	***F***	***P***	**ges**
**Vegetation**	2	72	24.079	9.84e-09	0.401
**Trophic group**	5	72	37.416	1.02e-18	0.722
**Vegetation*trophic group**	10	72	5.677	3.24e-06	0.441

Table S2 Results of Tukey's test applied to identify significant differences in soil nematode abundances (total) and richness (genera) between native open vegetation, open vegetation invaded by individuals of exotic *Pinus elliottii* and a plantation of *P. elliottii* in montane ecosystems of the Atlantic Forest domain, south-eastern Brazil (ANOVAs, Table S1 a,b). Gr, native open vegetation; PG, individuals of *P. elliottii* invading native open vegetation; Pi, *P. elliottii* plantation.

	**group1**	**group2**	**null.value**	**estimate**	**conf.low**	**conf.high**	***P*.adj**	***P*.adj.signif**
Abundance								
	Gr	PG	0	-1.380902	-13.69541	10.933600	0.9580	ns
	Gr	Pi	0	-16.115391	-28.42989	-3.800888	0.0088	**
	PG	Pi	0	-14.734489	-27.04899	-2.419986	0.0169	*
Richness								
	Gr	PG	0	-0.6	-4.372929	3.172929	0.90600	ns
	Gr	Pi	0	-5.8	-9.572929	-2.027071	0.00388	**
	PG	Pi	0	-5.2	-8.972929	-1.427071	0.00823	**

Table S3 Results of Tukey's test applied to identify significant differences in the abundance of different soil nematode trophic groups within native open vegetation (Gr), open vegetation invaded by individuals of exotic *Pinus elliottii* (PG), and a plantation of *P. elliottii* (Pi) in montane ecosystems of the Atlantic Forest domain, south-eastern Brazil (ANOVA, Table S1 c). Alg, algivore; Bac, bacterivore; Fun, fungivore; Omn, omnivore; Phy, phytophage; Pre, predator.

	**Vegetation**	**group1**	**group2**	**null.value**	**estimate**	**conf.low**	**conf.high**	**p.adj**	**p.adj.signif**
1	Gr	Alg	Bac	0	6.940626e+00	0.84037635	13.04087553	1.93e-02	*
2	Gr	Alg	Phy	0	1.957656e+01	13.47631017	25.67680936	8.12e-09	****
3	Gr	Alg	Fun	0	3.105272e+00	-2.99497795	9.20552123	6.22e-01	ns
4	Gr	Alg	Omn	0	5.085241e+00	-1.01500900	11.18549019	1.42e-01	ns
5	Gr	Alg	Pre	0	-2.665913e-01	-6.36684091	5.83365827	1.00e+00	ns
6	Gr	Bac	Phy	0	1.263593e+01	6.53568424	18.73618342	1.72e-05	****
7	Gr	Bac	Fun	0	-3.835354e+00	-9.93560389	2.26489529	4.02e-01	ns
8	Gr	Bac	Omn	0	-1.855385e+00	-7.95563494	4.24486425	9.32e-01	ns
9	Gr	Bac	Pre	0	-7.207217e+00	-13.30746685	-1.10696767	1.41e-02	*
10	Gr	Phy	Fun	0	-1.647129e+01	-22.57153772	-10.37103853	2.06e-07	****
11	Gr	Phy	Omn	0	-1.449132e+01	-20.59156876	-8.39106958	1.91e-06	****
12	Gr	Phy	Pre	0	-1.984315e+01	-25.94340068	-13.74290150	6.24e-09	****
13	Gr	Fun	Omn	0	1.979969e+00	-4.12028064	8.08021855	9.12e-01	ns
14	Gr	Fun	Pre	0	-3.371863e+00	-9.47211255	2.72838663	5.39e-01	ns
15	Gr	Omn	Pre	0	-5.351832e+00	-11.45208151	0.74841768	1.09e-01	ns
16	PG	Alg	Bac	0	5.358877e+00	-1.20162950	11.91938305	1.56e-01	ns
17	PG	Alg	Phy	0	1.250597e+01	5.94546138	19.06647393	5.91e-05	****
18	PG	Alg	Fun	0	1.492968e+00	-5.06753819	8.05347437	9.80e-01	ns
19	PG	Alg	Omn	0	2.786723e+00	-3.77378315	9.34722941	7.75e-01	ns
20	PG	Alg	Pre	0	-3.298258e-01	-6.89033210	6.23068046	1.00e+00	ns
21	PG	Bac	Phy	0	7.147091e+00	0.58658460	13.70759716	2.72e-02	*
22	PG	Bac	Fun	0	-3.865909e+00	-10.42641497	2.69459759	4.71e-01	ns
23	PG	Bac	Omn	0	-2.572154e+00	-9.13265992	3.98835263	8.27e-01	ns
24	PG	Bac	Pre	0	-5.688703e+00	-12.24920888	0.87180368	1.16e-01	ns
25	PG	Phy	Fun	0	-1.101300e+01	-17.57350584	-4.45249329	3.33e-04	***
26	PG	Phy	Omn	0	-9.719245e+00	-16.27975080	-3.15873825	1.50e-03	**
27	PG	Phy	Pre	0	-1.283579e+01	-19.39629976	-6.27528720	4.05e-05	****
28	PG	Fun	Omn	0	1.293755e+00	-5.26675123	7.85426132	9.89e-01	ns
29	PG	Fun	Pre	0	-1.822794e+00	-8.38330019	4.73771237	9.53e-01	ns
30	PG	Omn	Pre	0	-3.116549e+00	-9.67705523	3.44395732	6.86e-01	ns
31	Pi	Alg	Bac	0	3.633394e+00	-0.04427699	7.31106429	5.42e-02	ns
32	Pi	Alg	Phy	0	3.609904e+00	-0.06776709	7.28757419	5.65e-02	ns
33	Pi	Alg	Fun	0	1.480444e+00	-2.19722697	5.15811431	8.11e-01	ns
34	Pi	Alg	Omn	0	1.170726e+00	-2.50694438	4.84839690	9.19e-01	ns
35	Pi	Alg	Pre	0	-1.332268e-15	-3.67767064	3.67767064	1.00e+00	ns

36	Pi	Bac	Phy	0	-2.349010e-02	-3.70116074	3.65418054	1.00e+00	ns
37	Pi	Bac	Fun	0	-2.152950e+00	-5.83062062	1.52472066	4.78e-01	ns
38	Pi	Bac	Omn	0	-2.462667e+00	-6.14033803	1.21500324	3.35e-01	ns
39	Pi	Bac	Pre	0	-3.633394e+00	-7.31106429	0.04427699	5.42e-02	ns
40	Pi	Phy	Fun	0	-2.129460e+00	-5.80713052	1.54821076	4.90e-01	ns
41	Pi	Phy	Omn	0	-2.439177e+00	-6.11684793	1.23849335	3.45e-01	ns
42	Pi	Phy	Pre	0	-3.609904e+00	-7.28757419	0.06776709	5.65e-02	ns
43	Pi	Fun	Omn	0	-3.097174e-01	-3.98738805	3.36795322	1.00e+00	ns
44	Pi	Fun	Pre	0	-1.480444e+00	-5.15811431	2.19722697	8.11e-01	ns
45	Pi	Omn	Pre	0	-1.170726e+00	-4.84839690	2.50694438	9.19e-01	ns

Table S4 Results of Tukey's test applied to identify significant differences in the abundance of soil nematode trophic groups among native open vegetation (Gr), open vegetation invaded by individuals of exotic *Pinus elliottii* (PG), and a plantation of *P. elliottii* (Pi) in montane ecosystems of the Atlantic Forest domain, south-eastern Brazil (ANOVA, Table S1 c). Alg, algivore; Bac, bacterivore; Fun, fungivore; Omn, omnivore; Phy, phytophage; Pre, predator.

	Trophic group	group1	group2	null.value	estimate	conf.low	conf.high	p.adj	p.adj.signif
1	Alg	Gr	PG	0	-0.1829431	-2.648835	2.2829493	0.979000	ns
2	Alg	Gr	Pi	0	-1.0223869	-3.488279	1.4435055	0.529000	ns
3	Alg	PG	Pi	0	-0.8394438	-3.305336	1.6264486	0.646000	ns
4	Bac	Gr	PG	0	-1.7646922	-6.385028	2.8556434	0.580000	ns
5	Bac	Gr	Pi	0	-4.3296192	-8.949955	0.2907165	0.067000	*
6	Bac	PG	Pi	0	-2.5649269	-7.185263	2.0554087	0.334000	ns
7	Fun	Gr	PG	0	-1.7952466	-6.585556	2.9950631	0.591000	ns
8	Fun	Gr	Pi	0	-2.6472148	-7.437525	2.1430949	0.337000	ns
9	Fun	PG	Pi	0	-0.8519682	-5.642278	3.9383416	0.884000	ns
10	Omn	Gr	PG	0	-2.4814605	-6.199955	1.2370340	0.217000	ns
11	Omn	Gr	Pi	0	-4.9369012	-8.655396	-1.2184067	0.010500	*
12	Omn	PG	Pi	0	-2.4554407	-6.173935	1.2630539	0.224000	ns
13	Phy	Gr	PG	0	-7.2535352	-15.620726	1.1136552	0.092300	ns
14	Phy	Gr	Pi	0	-16.9890431	-25.356233	-8.6218527	0.000423	***
15	Phy	PG	Pi	0	-9.7355079	-18.102698	-1.3683176	0.023000	*
16	Pre	Gr	PG	0	-0.2461776	-2.575327	2.0829722	0.957000	ns
17	Pre	Gr	Pi	0	-0.7557956	-3.084945	1.5733543	0.671000	ns
18	Pre	PG	Pi	0	-0.5096180	-2.838768	1.8195318	0.831000	ns

Table S5 Results of testing for non-random patterns of the co-occurrence of nematode genera (all, phytophage) in three vegetation types in montane ecosystems of the Atlantic Forest domain, south-eastern Brazil. Gr, native open vegetation; PG, individuals of exotic *Pinus elliottii* invading open vegetation; Pi, plantation of *P. elliottii*. The test was made by using the ecospat.Cscore function of the 'ecospat' R package v4.1.2 (Broennimann, 2025). SES, standardised effect size.

	**Observed C-score**	**Simulated C-score**	**P-value lesser**	**P-value greater**	**SES**
All genera					
Gr	1.083	1.049	0.914	0.100	1.382
PG	1.132	1.138	0.477	0.580	-0.274
Pi	0.846	0.887	0.120	0.933	-1.249
Phytophage					
Gr	0.778	0.784	0.563	0.600	-0.090
PG	1.111	1.112	0.649	0.562	-0.020
Pi	0.952	0.925	0.882	0.386	0.474

References

Broennimann O 2018. ecospat: spatial ecology miscellaneous methods. R package ver. 3.0.
